# Editorial: Response/resistance to PD-1 axis inhibitors: focus on the tumor microenvironment

**DOI:** 10.3389/fimmu.2025.1720955

**Published:** 2025-10-20

**Authors:** Parmees Fazeli, Diba Yaghoubi, Ioannis Vathiotis, Arutha Kulasinghe, Thazin N. Aung

**Affiliations:** ^1^ Department of Pathology, Yale University School of Medicine, New Haven, CT, United States; ^2^ Department of Comparative Pathobiology, Purdue University College of Veterinary Medicine, West Lafayette, IN, United States; ^3^ Third Department of Internal Medicine, Sotiria Thoracic Diseases Hospital of Athens, National and Kapodistrian University of Athens, Athens, Greece; ^4^ Frazer Institute, Faculty of Medicine, The University of Queensland, Brisbane, QLD, Australia

**Keywords:** PD-1 - PD-L1 axis, tumor immune microenvironment, immunotherapy, PD-L2, tertiary lymphoid structures (TLS), response, resistance biomakers

Immunotherapy has transformed the therapeutic landscape of oncology, reshaping standards of care in non-small cell lung cancer (NSCLC) (1), gastrointestinal malignancies, hepatocellular carcinoma (2), and beyond (3). Despite remarkable clinical success, durable benefit is achieved only in a subset of patients. Understanding the determinants of response and resistance, identifying rational combinations, and managing toxicity remain urgent priorities. In this Research Topic, fourteen contributions, including original research, reviews, study protocols, case reports, and methodological advances, collectively advance our knowledge of immune checkpoint regulation, tumor immune microenvironment (TIME) biology, predictive biomarkers, and innovative therapeutic strategies. Together, they provide a timely snapshot of where the field stands and where it is headed.

Several papers in this Research Topic highlight the need to move beyond the traditional binary PD-1/PD-L1 paradigm. Zhu et al. demonstrated that PD-L2 functions as an independent immune checkpoint in Microsatellite Instability-High (MSI-H) Colon Cancer, with distinct spatial localization compared to PD-L1, suggesting that dual targeting of PD-L1 and PD-L2 may augment antigen presentation and restore immune activity. Importantly, this raises questions about whether PD-L2 testing should be incorporated into future biomarker panels in colorectal and potentially other cancers. Complementing this, Abdala-Saleh et al. revealed that (blockade of) PD-1 and CTLA-4 regulate a CXCL9/10-CXCR3-IFNγ positive feedback loop that potentiates effector and cytotoxic T-cell responses. Their findings provide a mechanistic explanation for the observation that patients with CXCL9/10 high tumors respond more favorably to immune checkpoint inhibitors (ICIs) and further suggest therapeutic potential for engineered CXCL9- or CXCL10-Fc fusion proteins. These studies in mouse melanoma models collectively argue that checkpoint biology is not static but interconnected, and that mechanistic dissection of immune crosstalk may unlock new avenues for therapeutic intervention.

Xu and Shao highlighted the synergy between immune and vascular modulation, reviewing how anti-angiogenic therapy remodels tumor vasculature to enhance immune infiltration, alleviate hypoxia, and diminish immunosuppressive signaling, thereby amplifying the efficacy of ICIs. Such strategies already have precedent, such as in the combination of atezolizumab with bevacizumab in hepatocellular carcinoma, as well as ivonescimab, a bispecific anti-PD-1 and anti-vascular endothelial growth factor (VEGF) antibody, as a potential first-line therapy for PD-L1-positive advanced NSCLC patients (4, 5). However, ongoing refinements underscore the principle that vascular normalization is not just supportive but mechanistically synergistic. Importantly, Xu and Shao‘s review places angiogenesis at the intersection of immune suppression, hypoxia, and therapeutic resistance, suggesting that durable benefit will require attacking multiple hallmarks of tumor biology simultaneously.

NSCLC remains the central arena of immunotherapy research and continues to drive both clinical advances and conceptual innovation. Yang et al. provided evidence that neoadjuvant immuno-chemotherapy in stage III NSCLC induces tertiary lymphoid structure (TLS) formation and reshapes immune composition, with increased TLS density correlating with benefit. This highlights TLSs as functional biomarkers, reflecting not only immune presence but also immune organization. In parallel, Wang et al. introduced the first randomized phase II trial (InTRist), testing induction therapy with anti–PD-1 toripalimab plus chemotherapy followed by chemoradiotherapy and PD-1 consolidation in patients with bulky locally advanced NSCLC, a design that seeks to overcome hypoxia-driven radio resistance and improve resectability. Such sequence-focused strategies may define the next frontier of multimodality treatment. Raskova Kafkova et al. synthesized current knowledge of NSCLC tumorigenesis and ICI misuse, emphasizing *PI3K/AKT/mTOR* signaling and highlighting nanobodies, affibodies, probodies and DARPINs as promising next-generation therapeutics. Extending beyond molecular pathways, Shimizu et al. showed that tumor *Akkermansia muciniphila* predicts ICI response in NSCLC patients with low PD-L1 expression, thus linking host-microbe interactions with therapeutic efficacy. Taniguchi et al. reported on CD38 expression in small cell lung cancer (SCLC), demonstrating its correlation with immunosuppressive markers and induction after chemo-immunotherapy, nominating CD38 as both a biomarker and potential target. Collectively, these studies confirm NSCLC as both a clinical driver and conceptual incubator for immunotherapy innovation.

Beyond NSCLC, studies have begun to explore rarer thoracic malignancies, areas that have historically lacked therapeutic breakthroughs. Dong et al. described a remarkable case of SMARCA4-deficient undifferentiated thoracic tumor treated successfully with tislelizumab plus chemotherapy underscoring the promise of checkpoint inhibition even in genomically aggressive tumors typically considered refractory. Such case reports, though anecdotal, provide critical insights into the responsiveness of rare tumors and generate hypotheses for future prospective trials.

Contributions in gastrointestinal and hepatic cancers highlight the breadth of immunotherapy’s reach. Yang et al. presented a case of pancreatic cancer achieving stabilization with toripalimab plus surufatinib, guided by TIME analysis. Du et al. applied next-generation sequencing to stratify advanced hepatocellular carcinoma patients, constructing a nomogram integrating PD-L1, tumor mutational burden, TERT, and TP53, thereby providing a framework for precision immunotherapy. Liu et al. extended this system prospectively by reporting that opioid-free anesthesia (OFA) shifts tumor-associated macrophage polarization toward an M1 phenotype in gastric cancer patients undergoing neoadjuvant PD-1 therapy, suggesting perioperative anesthetic choice as a modulator of immune outcomes. Together, these reports show that gastrointestinal cancers, often characterized by immunologically “cold” microenvironments, may be rendered more responsive by thoughtful integration of systemic and supportive interventions.

Methodological innovation was also represented. Azam et al. demonstrated that deep learning models trained with same-section ground truth labels outperform those trained on adjacent sections when applied to virtual staining for CD3^+^ T-cells in NSCLC, leading to more reliable prognostic stratification. This work highlights the critical importance of training data and establishes new benchmarks for clinically reliable artificial intelligence (AI)-driven pathology. As computational pathology becomes more deeply integrated into oncology, such advances will be critical to ensure that predictive tools translate into real-world patient benefit.

Finally, the challenge of toxicity was addressed by Wang et al., who explored the potential role of Traditional Chinese Medicine (TCM) in managing checkpoint inhibitor-induced colitis. With its capacity to modulate microbiota and inflammatory signaling, traditional medicine may complement conventional immunosuppression in resource-limited or culturally distinct settings. When considered alongside perioperative anesthesia studies, these reports illustrate that immunotherapy outcomes depend as much on systemic and supportive interventions as on direct tumor targeting.

Across these diverse studies, several unifying themes emerge. The TIME remains central: TLS formation in NSCLC, vascular normalization in digestive cancers, macrophage repolarization in gastric cancer, and microbiome-tumor interactions collectively underscore that immune contexture, not tumor-intrinsic features alone, dictates therapeutic response. Combination strategies continue to dominate the landscape, such as rational integration of PD-1 blockade with chemotherapy, tyrosine kinase inhibitors, radiotherapy, anti-VEGF agents, or even anesthesia modulation consistently produces additive or synergistic effects. Precision medicine is advancing rapidly, with biomarkers such as PD-L2, CD38, *Akkermansia muciniphila*, and AI-derived immune phenotypes offering new dimensions of stratification beyond PD-L1 alone. Meanwhile, checkpoint biology itself is being redefined through deeper mechanistic insights into PD-L2, CTLA-4, and *CXCR3* signaling. Importantly, toxicity management is evolving in parallel, with integrative strategies, including TCM and perioperative optimization, broadening the therapeutic window.

In summary, this Research Topic highlights the extraordinary breadth of ongoing work at the intersection of immunotherapy and TIME research. Collectively, these fourteen papers chart a course toward more precise, durable, and tolerable treatments. They emphasize that immunotherapy now extends beyond the tumor bed to encompass host-microbiome interactions, perioperative care, anesthesia management, and computational pathology pipelines. The optimal deployment of immunotherapy will thus demand a holistic framework that integrates tumor biology, systemic physiology, host-microbe dynamics, and technological innovation. Guided by these insights, the future of cancer therapy will increasingly harness the full complexity of the immune system, delivering treatments that are more precise, more effective, and more patient-centered.
